# Analysis of status and influencing factors of knowledge, attitudes, and expectations towards assisted reproductive technology among infertile women in Lebanon: A cross-sectional study

**DOI:** 10.1371/journal.pone.0331989

**Published:** 2026-06-18

**Authors:** Reva Mosleh, Hiba Assi, Jana Kassir, Hassan Ajami, Joseph Azoury, Roula Ajrouche, Amal Al-Hajje

**Affiliations:** 1 Doctoral School of Science and Technology, Lebanese University, Hadath, Lebanon; 2 Clinical and Epidemiological Research Laboratory, Faculty of Pharmacy, Lebanese University, Hadath, Lebanon; 3 Hope Clinic, Al Zahraa Hospital University Medical Center, Beirut, Lebanon; 4 Azoury In Vitro Fertilization Clinic, Mount Lebanon Hospital, Beirut, Lebanon; 5 INSPECT-LB (Institut National de Santé Publique, d’Epidémiologie Clinique et de Toxicologie-Liban), Beirut, Lebanon; Medical Park Minatomirai, JAPAN

## Abstract

**Background:**

Infertility affects millions globally, and while assisted reproductive technology (ART) has become a cornerstone of its treatment, there is limited research on how Lebanese women perceive these services. This study aims to fill the gap by assessing each of the knowledge, attitudes, and expectations of Lebanese women experiencing difficulty conceiving towards ART.

**Methods:**

A cross-sectional study was conducted between June and September 2024, involving 346 Lebanese women from two fertility centers in Beirut. Participants were selected by simple random sampling to complete the questionnaire through individual interviews. Descriptive and bivariate analyses were performed, and generalized linear models were used to explore the associated factors of knowledge, attitudes, and expectation scores.

**Results:**

The results showed that 56.4% of participants had good knowledge, 54.9% had positive attitudes, and 73.7% exhibited high expectations. The generalized linear models revealed that previous ART use (β = 0.151), receiving ART information from a doctor (β = 0.064), female age (β = 0.005), and physical exercise (β = 0.119) were linked to higher knowledge, while higher family income (β = −0.133), history of immunodeficiency (β = −0.275), living in centers (β = −0.066), were linked to lower knowledge. For attitudes, residing in South Lebanon (β = 6.136), having a history of ovarian cyst removal (β = 2.065), receiving ART information from a doctor (β = 1.151) and female age (β = 0.120) were linked to positive attitudes, while living in centers (β = −1.835), having a regular menstrual period (β = −1.521) were linked to lower attitudes. For the expectations, female hormonal disorder (β = 2.758) was significantly associated with higher expectations, while advanced female age (β = −0.768) was associated with lower expectations.

**Conclusion:**

This study identifies associated factors influencing knowledge, attitudes, and expectations toward ART. To optimize these aspects, interventions should focus on effective education, personalized treatments, reducing barriers, and providing psychological support to improve women’s reproductive health. Further research is needed to explore these factors in the broader population.

## Introduction

Infertility impacts millions of individuals of childbearing age worldwide. It is estimated that approximately 48 million couples and 186 million people globally are living with infertility [1]. Based on global-metrics data sets, in 2025, Lebanon’s fertility rate stands at 1.99 births per woman, marking a 0.6 decline from 2024, and projections indicate a downward trend until 2050 [2]. The World Health Organization (WHO) defines infertility as a disease of the male or female reproductive system, characterized by the failure to achieve a pregnancy after 12 months or more of regular unprotected sexual intercourse [1]. The causes of infertility are equally divided between both genders, with male and female factors each contributing to around 35% of cases. In 20% of cases, a combination of both factors is involved, while 10% remain unexplained [3]. The management of infertility has evolved over the past four decades to include ART, which was a revolutionary in the study of medicine. The WHO, in collaboration with various societies such as the American Society for Reproductive Medicine, the European Society for Human Reproduction and Embryology and others, has standardized numerous definitions used in medically assisted reproduction [4]. ART is defined as any treatment or procedure that involves the in vitro handling of human oocytes, sperm, or embryos to establish a pregnancy [4]. This encompasses techniques such as in vitro fertilization (IVF), intracytoplasmic sperm injection (ICSI), preimplantation genetic testing (PGT), and others, but doesn’t include intrauterine insemination (IUI) [4]. There are several indications for the use of ART in both men and women, including tubal factor infertility, diminished ovarian reserve, ovulatory dysfunction, oligospermia or azoospermia, and unexplained infertility [5]. Fortunately, fertility treatment centers are growing rapidly in the Middle East, outpacing development in Europe and North America. For instance, Iran has over 70 IVF centers, and Turkey has more than 110, along with active centers in the United Arab Emirates, Egypt, Jordan, and Saudi Arabia [6]. Additionally, lower treatment costs make infertility services more accessible in the Middle East [7]. In Lebanon, there is no specific law or regulation specially dedicated to the reproduction practices. Provisions within the 1994 law of medical ethics permit artificial insemination or pregnancy by using assisted fertility techniques for married couples [8]. Despite the ongoing widespread use of assisted reproduction across the world, previous studies indicate that women have misconceptions about ART [9,10]. For instance, an Iranian study shows that about 63.8% of the group studied had poor knowledge, 26.9% had fair knowledge, and less than 10% had good knowledge [11]. Other research shows that public attitudes in the United States of America and many other Western countries toward ART are positive [12,13]. When it comes to the expectations, a Brazilian study found that 42% of women seeking fertility treatment expected their chances of pregnancy after a single IVF cycle to be over 60% [14]. Although Lebanon lacks comprehensive data on the utilization of assisted reproduction, there is a noticeable presence of well-established IVF centers, and many individuals and couples are pursuing IVF treatments. This is supported by a cross-sectional study published in 2020, which surveyed over 600 Lebanese individuals regarding their knowledge and perceptions of infertility. The study found that 93.9% of respondents believe infertility should be medically treated, 92.3% think it is socially acceptable to seek medical fertility treatment, 82.8% find it acceptable to use IVF if infertile, and 66.2% believe that using IVF is acceptable according to their religion [15]. Nevertheless, previous research in Lebanon shows that women’s fertility quality of life is lower compared to Western countries, influenced by factors such as difficulties in conceiving, not having children, and facing societal stigma [16].

Given the increasing prevalence of infertility and broader access to ART services in Lebanon, there is a noticeable gap in research regarding how Lebanese women perceive these treatments. This study, therefore, aims to assess the knowledge, attitudes, and expectations of Lebanese women experiencing difficulty conceiving with regard to ART, and explore the associated factors that influence each.

## Methodology

### Study design and data collection

This cross-sectional study was conducted from June 1, 2024, to September 30, 2024, at Hope Clinic (Al Zahraa Hospital University Medical Center), a private university-affiliated teaching hospital, and Azoury IVF Clinic (Mount Lebanon Hospital), a private referral center—both located in Beirut. A simple random sampling method was employed; patients were selected from those seen by participating clinicians. Eligible individuals were approached and invited to participate, and those who consented were individually interviewed by three researchers—one PhD candidate and two Master’s students in Clinical Research and Pharmacoepidemiology using a structured paper questionnaire. Participation was anonymous and voluntary, with written consent obtained. To ensure anonymity, consent forms were stored separately from the completed questionnaires, which contained no personal identifiers. The three interviewers coordinated through regular team meetings throughout the data collection period to discuss any discrepancies and ensure consistency in interviewing techniques. Interviews were conducted in Arabic and took 12–15 minutes to complete.

### Inclusion and exclusion criteria

The study included Lebanese married women of reproductive age (≥18 years), actively attempting to conceive, and diagnosed with infertility regardless of the infertility source. Infertility was defined as the inability to conceive after one year of unprotected intercourse. Exclusion criteria were women with incomplete questionnaires (any questionnaire with missing responses in any section).

### Sample size

The sample size was calculated using the standard formula for estimating a proportion in a finite population survey through Epi Info software [17]. The formula used:

$n=\frac{N\times Z^{\text{2}}\times p\times (\text{1}-p)}{d^{\text{2}}\times (N-\text{1})+Z^{\text{2}}\times p\times (\text{1}-p)}$

N is the total Lebanese population size in 2024 (N = 5,800,000), z is the z-score corresponding to the desired confidence level (1.96 for 95% confidence level), d = 0.05 margin of error, and an expected infertility prevalence of 0.343, resulted in a target sample size of 346 participants [18,19].

### Study tool

The questionnaire was developed following a comprehensive review of the literature and was structured into three main sections. The first section included 23 questions addressing socio-demographic (residence, medical insurance, income, educational level, etc.) and lifestyle factors (smoking, alcohol consumption, physical exercise, etc.) [20]. The second section included 16 questions on health status, covering chronic conditions (hypertension, diabetes, dyslipidemia, etc.), gynecological history (menstruation, history of pregnancy, history of gynecological surgery, etc.), and infertility history (e.g., causes, family history) [21–24]. The third section contained 10 questions assessing knowledge of ART (with responses of “Yes,” “No,” or “I Don’t Know”), 12 questions evaluating attitudes towards ART (using a Likert scale), and 9 questions examining expectations related to ART (with responses of “Yes,” “No,” or “I Don’t Know”) [11,25–28]. The questionnaire was translated from English to Arabic and back-translated to ensure linguistic accuracy. To establish face and content validity, the tool was reviewed by two experts in the field of infertility, including an Obstetrics and Gynecology reproductive specialist and a maternal health researcher. It was pilot tested among 10–15 participants, which led to minor revisions.

### Data analysis

The data from the questionnaire were analyzed using SPSS version 26, with descriptive statistics, bivariate, and multivariate analysis. Frequencies were calculated for categorical variables, and means and standard deviations were used for continuous variables. The normality of continuous variables was assessed both graphically and statistically through skewness and kurtosis [29]. Bivariate analysis of qualitative variables was conducted using the independent samples t-test or one-way ANOVA with normally distributed scores, and the Kruskal-Wallis or Mann-Whitney U test with non-normally distributed scores. For continuous variables, Pearson correlation was used for normal data, and Spearman correlation for non-normal data.

A Generalized Linear Model (GLM) was employed because the dependent variable, which is the knowledge score, was continuous but not normally distributed, which violates the assumptions of ordinary linear regression. The GLM also provided flexibility in handling multiple categorical independent variables. For the knowledge score outcome, a gamma distribution with a log link function was selected to account for the skewed distribution of the data, while attitudes and expectations scores were analyzed using a linear scale due to their normal distribution. Model adequacy and fit were further evaluated through the examination of residual plots. It allows for the identification of significant associated factors for each of the knowledge, attitudes and expectations scores, including variables with p-values <0.2. P-values <0.05 were considered statistically significant. Cronbach’s alpha was used to assess the internal consistency of the scores. While Cronbach’s alpha values of 0.7 and above are recommended [30], a value of 0.6 or higher can still be acceptable, especially in exploratory studies and social sciences research [31]. In this study, items with a Cronbach’s alpha of 0.6 or above were considered to have acceptable reliability. The alpha values for the knowledge, attitudes, and expectations scores were 0.63, 0.68, and 0.78, respectively.

### Scoring

Individual scores for knowledge, attitudes, and expectations related to ART were calculated and used as dependent variables in analyses. As no standardized cut-off values exist for these scores, the cut-off points for each were determined post hoc based on the sample median. The 10-question knowledge score was scored 1 for correct answers and 0 for incorrect or “I don’t know” responses, with a range of 0–10. Scores 0–8 indicated low to moderate knowledge, and 9–10 indicated high knowledge. The attitudes score was based on a 12-question Likert scale from 1 (“Strongly Disagree”) to 5 (“Strongly Agree) ranging from 12 to 60. Scores <49 indicated negative attitudes, and those ≥50 indicated positive attitudes. The 9-question expectations score was coded 1 for “yes” and 0 for “no” or “don’t know,” with a range of 0–9. Scores <5 indicated negative expectations, and those ≥5 indicated positive expectations.

### Ethical consideration

This study employed a questionnaire for data collection, without invasive procedures or interventions. The study protocol, survey, and consent forms were reviewed and approved by the Institutional Review Board of the School of Pharmacy at the Lebanese University (7/24/D) adhered to the Declaration of Helsinki. Since the participating centers are private clinics without independent IRBs, the University’s IRB approval was accepted by these clinics to cover all study sites. Data was anonymous, non-identifiable, and stored according to the University’s data protection guidelines. Written informed consent was obtained, and participants were informed that their involvement was voluntary and they could withdraw at any time without justification.

## Results

### Sociodemographic characteristics and health status of the participants

The study included 346 women, with a mean age of 34 years (± 6.48 years). Of these, 53.47% were from Azoury clinic, and 46.5% were from Hope clinic. Half of the participants are workers, 53.47% lived in central areas, and 57.51% had health insurance. Additionally, 90.46% were non-cigarette smokers, 63.29% were non-nargileh smokers, and 88.44% non-alcohol consumers. For the past medical history, 38.2% had at least one chronic disease. Among these, obesity (53.78%) and thyroid disorders (25%) were the most common. Among the participants, 34.39% had routine Pap smears, and 52.02% had at least one previous gynecological surgery. Among women with a history of gynecological surgery, cesarean section (38.89%) and laparoscopy (36.67%) were the most frequent. Difficulty conceiving was primarily due to female factors (41.91%), such as Polycystic Ovary Syndrome (PCOS) (36.56%), ovulatory disorders (33.10%), and endometriosis (28.97%). Among those who reported infertility due to male factors, low sperm count (93.75%) and low sperm quality (92.50%) were common issues. Also, 9.83% had a family history of infertility, predominantly on the mother’s side (52.94%) (Table 1).

**Table 1:** Sociodemographic characteristics and health status of the participants (N = 346).

Variable	Frequency (%)	Mean ± SD
**Age (years)**		33.82 ± 6.48
**BMI (kg/m**^**2**^)		26.19 ± 4.77
**Region**		
Mount Lebanon	113 (32.66)	
Beirut	85 (24.57)	
Beqaa	64 (18.50)	
South	46 (13.29)	
North	30 (8.67)	
Nabatiyeh	8 (2.31)	
**Family monthly income**		
< 250 USD	68 (19.65)	
> 250–500 USD	119 (34.39)	
> 500–1000 USD	64 (18.50)	
> 1000–2000 USD	35 (10.12)	
> 2000 USD	60 (17.34)	
**Educational level**		
Didn’t receive any education	4 (1.16)	
Elementary or Intermediate	42 (12.14)	
High School	61 (17.63)	
University Degree	180 (52.02)	
Post-Graduate Degree	59 (17.05)	
**Past medical history of chronic disease**	132 (38.20)	
**Infertility cause**		
Female factors	145 (41.90)	
Male factors	80 (23.10)	
Combined factors	50 (14.50)	
Unexplained factors	71 (20.50)	

Abbreviations: BMI = Body Mass Index, N = Frequency, SD = Standard Deviation, USD = United States Dollar.

### Description of the scores

#### Knowledge score.

The results showed a mean knowledge score of 8.36 ± 1.91 (median = 9; range = 0–10). Of the participants, 195 (56.4%) demonstrated high knowledge, while 151 (43.6%) had moderate to low knowledge (Table 2).

**Table 2:** Women’s responses to knowledge items on ART.

Knowledge about ART	No N (%)	Yes N (%)	I don’t know N (%)
Have you heard about ART?	11 (3.10)	332 (96.00)	3 (0.90)
Have you heard about IVF?	9 (2.60)	335 (96.80)	2 (0.60)
Does IVF take place in the laboratory?	26 (7.50)	276 (79.80)	44 (12.70)
Are ovulation induction and egg aspiration important steps prior to IVF?	6 (1.70)	306 (88.50)	34 (9.80)
Have you heard about ICSI?	85 (24.50)	241 (69.70)	20 (5.80)
Do you know that ICSI is a laboratory process where the sperm is injected directly into the egg?	50 (14.50)	215 (62.10)	81 (23.40)
Have you heard about IUI?	75 (21.70)	252 (72.80)	19 (5.50)
Do you know that eggs can be frozen?	3 (0.90)	339 (98.00)	4 (1.10)
Do you know that sperms can be frozen?	28 (8.10)	289 (83.50)	29 (8.40)
Do you know that embryos can be frozen?	16 (4.60)	306 (88.40)	24 (7.00)

#### Attitudes score.

The results showed a mean attitude score of 49.5 ± 5.43 (median = 50; range = 12–60). Of the participants, 190 (54.9%) demonstrated positive attitudes, while 156 (45.1%) demonstrated negative attitudes (Table 3).

**Table 3:** Women’s responses to attitudes items on ART.

Attitude about ART	Strongly disagree N (%)	Disagree N (%)	Neutral N (%)	Agree N (%)	Strongly agree N (%)
If you and your partner have difficulty conceiving, how much do you agree to seek ART?	2 (0.60)	5 (1.50)	7 (2.00)	43 (12.40)	289 (83.50)
How much do you agree that ART is accepted by our community?	2 (0.60)	9 (2.60)	24 (6.90)	110 (31.80)	201 (58.10)
How much do you agree that ART babies are accepted in our community?	3 (0.90)	2 (0.60)	9 (2.60)	74 (21.30)	258 (74.60)
How much do you agree that ART is accepted by religion?	6 (1.70)	7 (2.00)	32 (9.30)	92 (26.60)	209 (60.40)
How much do you agree that ART gives hope to couples having difficulty conceiving?	1 (0.30)	2 (0.60)	7 (2.00)	48 (13.90)	288 (83.20)
How much do you agree with a female freezing her eggs to achieve a future pregnancy?	4 (1.10)	11 (3.20)	19 (5.50)	56 (16.20)	256 (74)
How much do you agree with a male freezing his sperm to achieve a future pregnancy?	4 (1.10)	11 (3.20)	39 (11.30)	57 (16.50)	235 (67.90)
How much do you agree that a couple can freeze embryos to achieve a future pregnancy?	8 (2.30)	13 (3.80)	29 (8.40)	60 (17.30)	236 (68.20)
To what extent do you agree with the idea of donating embryos, sperm, or eggs?	131 (37.90)	76 (22.00)	46 (13.30)	41 (11.80)	52 (15.00)
To what extent do you encourage other couples to seek ART	3 (0.90)	0 (0.00)	3 (0.90)	49 (14.20)	291 (84.00)
How much do you agree with the idea of gestational surrogacy?	133 (38.40)	92 (26.60)	47 (13.60)	45 (13.00)	29 (8.40)
Do you agree for a couple to tell their surroundings that they are seeking ART?	48 (13.90)	71 (20.50)	42 (12.10)	75 (21.70)	110 (31.80)

#### Expectations towards ART.

The results showed a mean expectation score of 5.12 ± 1.55 (median = 5; range = 0–9). Of the participants, 255 (73.7%) demonstrated high expectations, while 91 (26.3%) demonstrated low expectations (Table 4).

**Table 4:** Women’s responses to expectations items on ART.

Expectations about ART	No N (%)	Yes N (%)	I don’t know N (%)
Do you expect that ART procedures will always be easy?	278 (80.40)	45 (13.00)	23 (6.60)
Do you expect that ART procedures will always be successful?	322 (93.10)	7 (2.00)	17 (4.90)
Do you expect that ART procedures are accessible and affordable in our country?	274 (79.20)	53 (15.30)	19 (5.50)
Do you expect that the chances of getting pregnant by ART are higher when the female is younger?	53 (15.30)	270 (78.00)	23 (6.70)
Do you expect that a couple will try again even if the ART pregnancy has failed before?	17 (5.00)	323 (93.40)	6 (1.70)
Do you expect that getting pregnant with ART is the same as conceiving naturally?	91 (26.30)	220 (63.60)	35 (10.10)
Do you expect that ART procedures are safe for women?	39 (11.30)	256 (74.00)	51 (14.70)
Do you expect that ART procedures are safe for the fetus?	23 (6.60)	272 (78.70)	51 (14.70)
Do you expect that an ART procedure increases the likelihood of getting pregnant with twins?	10 (2.90)	327 (94.50)	9 (2.60)

### Bivariate analysis

#### Knowledge score.

Higher mean ART knowledge scores were significantly associated to living in rural areas (p = 0.009), exercising (p = 0.028), a history of ART use (p < 0.001), gynecological surgery (p = 0.015), laparoscopy (p = 0.035), and learning about ART from a doctor (p = 0.009) or the internet (p = 0.013). Longer marriage duration also correlated positively with knowledge (p = 0.007). Conversely, more frequent nargileh use was negatively associated with ART knowledge (p = 0.009) (Table 5).

**Table 5:** Bivariate analysis of factors associated with knowledge score towards ART.

Qualitative variables	Mean rank*	p-value
Living area		0.009
Rural	188.12	
Center	160.78	
Physical exercise (Y)	189.93	0.028
History of ART utilization (Y)	196.23	<0.001
History of gynecological surgery (Y)	185.65	0.015
History of laparoscopy (Y)	196.00	0.035
Heard about ART from the doctor (Y)	186.01	0.009
Heard about ART from the internet (Y)	189.39	0.013
**Quantitative variables**	**Rs**	**p-value**
Duration of marriage	0.14	0.007
Number of nargileh sessions/week	−0.14	0.009

* Mann-Whitney U test.

Rs = Spearman correlation coefficient, Y = Yes.

Variables that lacked significance with the knowledge score (Azoury/hope clinic p = 0.659, age p = 0.187, BMI p = 0.855, residence p = 0.71, monthly income p = 0.135, cigarette smoking p = 0.177, nargileh smoking p = 0.102, educational level p = 0.091, health insurance p = 0.919, occupation p = 0.712, chronic disease p = 0.925).

#### Attitudes score.

Higher mean attitude scores were significantly associated with living in rural areas (p = 0.028), higher education level (p = 0.023), having health insurance (p = 0.025), and a history of gynecological surgery (p = 0.001), cesarean section (p = 0.012), and laparoscopy (p = 0.004). Additionally, regular menstrual periods (p = 0.01), previous pregnancies (p = 0.006), ART utilization history (p = 0.001), hearing about ART from the doctor (p = 0.017), and recognizing advanced female age as a cause of infertility (p = 0.038) were also linked to higher attitude scores. Age (p < 0.001), weight (p = 0.02), BMI (p = 0.048), and duration of marriage (p = 0.044) showed positive correlations with attitude scores (Table 6).

**Table 6:** Bivariate analysis of factors associated with attitudes score towards ART.

Qualitative variables	Mean* ± SD	p-value
Living area		0.028
Rural	50.20 ± 5.16	
Center	48.90 ± 5.60	
Educational level		0.023**
Didn’t receive any education	53 ± 2.50	
Elementary or intermediate	47.80 ± 5.95	
High school	49.70 ± 4.97	
University degree	49.30 ± 5.80	
Post-graduate degree	51 ± 3.80	
Having medical or health insurance (Y)	50 ± 4.90	0.025
History of gynecological surgery (Y)	50.50 ± 4.70	0.001
History of cesarean section (Y)	51 ± 4.22	0.012
History of laparoscopy (Y)	51.20 ± 4.30	0.004
Regular menstrual period (Y)	49.97 ± 5.30	0.01
History of previous pregnancy (Y)	50.34 ± 4.90	0.006
History of ART utilization (Y)	50.35 ± 4.88	0.001
Advanced female age as an infertility cause (Y)	51.70 ± 4.53	0.038
Heard about ART from the doctor (Y)	50.14 ± 5.35	0.017
**Quantitative variables**	**R**	**p-value**
Age	0.22	<0.001
Weight	0.12	0.02
BMI	0.10	0.048
Duration of marriage	0.10	0.044

* Independent samples t-test and One-way ANOVA.

** Post-hoc analysis: Educational level (post-graduate vs elementary or intermediate p = 0.026, post-graduate vs university degree p = 0.237, post-graduate vs high school p = 1, post-graduate vs no education p = 1).

R = Pearson correlation for normally distributed variables and Spearman correlation for non-normally distributed ones.

Variables that lacked significance with the attitudes score (Azoury/hope clinic p = 0.327, exercising p = 0.745, cigarette smoking p = 0.697, nargileh smoking p = 0.944, alcohol consumption p = 0.307, residence p = 0.15, occupation p = 0.175).

#### Expectations score.

Higher mean expectations scores were significantly associated with higher family income (p = 0.003), different fertility clinics (p < 0.001), a history of gynecological surgery (p = 0.028), cesarean section (p = 0.003), regular menstruation (p = 0.044), and ART utilization history (p = 0.002). Participants with no history of hypertension (p = 0.044) and visual problems (p = 0.008) were linked to higher expectations scores. Besides, BMI (p = 0.021) showed a negative correlation with expectations towards ART (Table 7).

**Table 7:** Bivariate analysis of factors associated with expectations scores towards ART.

Qualitative variables	Mean* ± SD	p-value
Family income		0.003**
<250 USD	4.68 ± 1.67	
>250–500 USD	4.94 ± 1.71	
>500–1000 USD	5.40 ± 1.20	
>1000–2000 USD	5.30 ± 1.34	
>2000 USD	5.62 ± 1.33	
Name of the clinic		<0.001
Azoury	5.55 ± 1.23	
Hope	4.64 ± 1.72	
History of gynecological surgery (Y)	5.30 ± 1.55	0.028
History of cesarean section (Y)	5.60 ± 1.22	0.003
Regular menstruation (Y)	5.23 ± 1.51	0.044
History of hypertension (N)	5.14 ± 1.53	0.044
History of visual problems (N)	5.16 ± 1.53	0.008
History of ART utilization (Y)	5.34 ± 1.45	0.002
**Quantitative variables**	**Rp**	**p-value**
BMI	−0.12	0.021

* Independent samples t-test and One-way ANOVA.

** Post-hoc analysis: family income (<250 vs > 2000 USD p = 0.005, 250–500 vs > 2000 USD p = 0.048, > 500–1000 vs > 2000 USD p = 1, > 1000–2000 vs > 2000 USD p = 1).

Rp = Pearson correlation.

Variables that lacked significance with the expectations score (age p = 0.709, duration of marriage p = 0.082, residence p = 0.146, educational level p = 0.245, cigarette smoking p = 0.558, nargileh smoking p = 0.123, exercising p = 0.206, health insurance p = 0.495, living area p = 0.238, occupation p = 0.51).

### Multivariate analysis

The results from the first GLM, with the knowledge score as the dependent variable, showed that having a history of previous ART utilization increases the knowledge score by 0.151 points. Similarly, receiving information about ART from a doctor (β = 0.064), female age (β = 0.005), and physical exercise (β = 0.119) were positively associated with higher knowledge scores. On the other hand, factors such as higher family income (β = −0.133), a medical history of immunodeficiency (β = −0.275), living in centers (β = −0.066), and more frequent exercise per week (β = −0.028) were significantly associated with lower knowledge scores.

The second GLM analysis, with attitudes score as the dependent variable, showed that residing in South Lebanon (β = 6.136), North Lebanon (β = 4.624), Bekaa (β = 5.359), Beirut (β = 5.321), and Mount Lebanon (β = 4.979) were significantly associated with higher attitudes scores compared to residing in Nabatiyeh. Additionally, having a history of ovarian cyst removal (β = 2.065), receiving information about ART from a doctor (β = 1.151), and female age (β = 0.120) were also linked to more positive attitudes towards ART. In contrast, living in centers (β = −1.835), having a regular menstrual period (β = −1.521), and having a medical history of asthma/COPD (β = −3.781), gastric ulcers (β = −3.947), or epilepsy (β = −5.192) were significantly associated with lower attitudes towards ART.

The third GLM analysis, with expectations score as the dependent variable, revealed that reporting hormonal disorders as a cause of female infertility (β = 2.758) was significantly associated with higher expectations regarding ART. In contrast, advanced female age (β = −0.768) and being a patient at the Hope clinic (β = −0.7) were significantly associated with lower expectations (Table 8).

**Table 8:** Multivariate analysis.

Associated factors	Coefficient β	SE	95% CI		p-value
			Lower	Upper	
**Model 1: Generalized linear model with gamma log link taking the knowledge score as the dependent variable**					
History of ART utilization	0.151	0.0304	0.091	0.210	<0.001
Heard about ART from the doctor	0.064	0.0314	0.002	0.125	0.042
Family income >1000–2000 USD	−0.133	0.06	−0.251	−0.016	0.027
Having a medical history of immunodeficiency	−0.275	0.0967	−0.465	−0.085	0.004
Living in centers	−0.066	0.0277	−0.12	−0.012	0.017
Age	0.005	0.0025	0.001	0.01	0.028
Physical exercise	0.119	0.051	0.019	0.219	0.02
Frequency of exercise per week	−0.028	0.0122	−0.052	−0.004	0.022
**Model 2: Generalized linear model taking the attitudes score as the dependent variable**					
Living in centers	−1.835	0.6274	−3.065	−0.606	0.003
Residence*					
South Lebanon	6.136	1.8757	2.459	9.812	0.001
North Lebanon	4.624	1.9420	0.818	8.431	0.017
Bekaa	5.359	1.8125	1.807	8.912	0.003
Beirut	5.321	1.8792	1.638	9.004	0.005
Mount Lebanon	4.979	1.8166	1.418	8.539	0.006
Having a regular menstrual period	−1.521	0.6064	−2.709	−0.332	0.012
History of ovarian cyst removal	2.065	1.0307	4.086	4.016	0.045
Heard about ART from a doctor	1.151	0.5401	0.093	2.210	0.033
Medical history of asthma or COPD	−3.781	1.8102	−7.329	−0.233	0.037
Medical history of gastric ulcers	−3.947	1.6761	−7.232	−0.662	0.019
Medical history of epilepsy	−5.192	2.4996	−10.091	−0.293	0.038
Age	0.12	0.0506	0.021	0.219	0.017
**Model 3: Generalized linear model taking the expectations score as the dependent variable**					
Hope clinic	−0.7	0.2555	−1.201	−0.199	0.006
Female hormonal disorders (as an infertility cause)	2.758	1.1576	0.489	5.027	0.017
Advanced female age (as an infertility cause)	−0.768	0.3755	−1.504	−0.032	0.041

*Reference residence = Nabatiyeh.

### Correlation between the knowledge, attitudes and expectations scores

**Fig 1:**
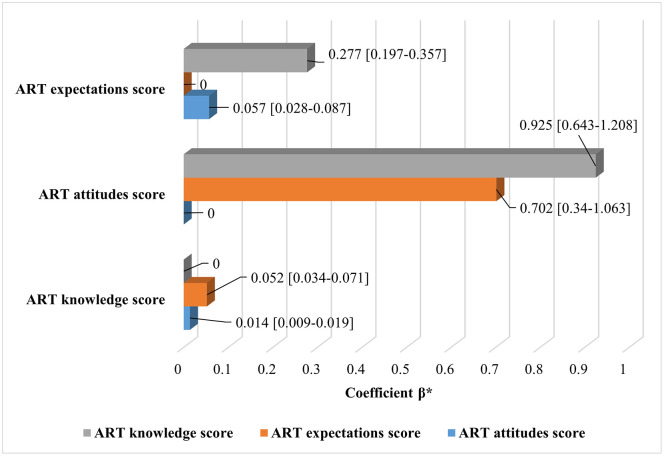
Correlation between the knowledge, attitudes, and expectations scores. * GLM with a linear scale for normally distributed scores and a gamma log link for non-normally distributed ones.

Positive attitudes and higher expectations are significantly correlated with higher knowledge scores on ART (p < 0.001). Also, higher knowledge and expectations are significantly correlated with more positive attitudes towards ART (p < 0.001). Also, higher knowledge and attitudes are significantly correlated with greater expectations towards ART (p < 0.001) (Fig 1.).

## Discussion

The study found that more than half of the participants had good knowledge of ART, similar to studies in Iran and contrary to studies in Egypt and Nigeria, which reported lower levels of knowledge [28,32,33]. These differences may be attributed to variations in cultural, educational, and healthcare access factors across different regions. More than half of the women had heard of various assisted reproductive procedures, including IVF, ICSI, and IUI, and the majority accurately defined IVF and ICSI. However, a higher proportion of participants correctly defined IVF compared to those who defined ICSI. It could be because the terminology for IVF is more widely discussed and recognized than ICSI. These results contrast with a study in Egypt, which found that most infertile couples provided incorrect definitions of both ICSI and IVF procedures [32]. When testing for the associated factors of increased knowledge, it was found that both a history of previous ART use and receiving information from a doctor were associated with greater knowledge. It may be because individuals with prior ART experience have gained more firsthand understanding of the procedures, while those who received information from a doctor benefited from accurate, reliable, and detailed explanations about ART. Advanced female age was positively associated with higher knowledge scores, which aligns with a study conducted in Iran [28]. Although the effect size was small (β = 0.005), this association may reflect that older women are more likely to have experienced infertility or concern about approaching pre-menopause age, leading to a deeper understanding of reproductive technologies. Unlike living in centers, residing in rural areas was associated with higher knowledge in contrast to other findings [28], possibly because fertility and family building may hold greater cultural significance among Lebanese women living in these areas. Also, those who engaged in physical exercise had greater knowledge compared to those who didn’t, likely due to their interest in health. Lifestyle factors have a significant impact on reproductive health [20]. While both exercise and frequency of exercise showed a positive association with knowledge score in bivariate testing, the multivariate model demonstrated contradictory directions. This shift likely reflects the adjustment for other covariates within the multivariate model, which can reveal confounding or suppressor effects that are not visible in the bivariate analyses. Additionally, the clinical effect of exercise frequency appears minimal (β = −0.028), suggesting limited practical significance despite statistical findings. Also, an unexpected inverse association existed between family income (>1000–2000 USD) and the knowledge score, and could be possibly explained by the fact that women with higher incomes may rely more heavily on healthcare providers and private consultations for information. Yet, those with lower income may be less accessible to infertility services and thus engage more actively in seeking information.

The findings indicate that attitudes towards ART among Lebanese women are generally positive, which is consistent with studies conducted in Egypt and Europe, but contrasts with the Nigerian study [32,34,35]. The positive attitude may be attributed to the fact that a significant number of ART treatments successfully address couples’ infertility, offering hope for having a child. The majority of respondents strongly agreed that they would seek ART and that ART-conceived children are accepted in society, aligning with studies conducted in Nigeria and Ghana [27,33]. In the context of cryopreservation, the majority strongly supported freezing eggs, sperm, and embryos for future use. However, most respondents rejected the concepts of gamete donation and gestational surrogacy. These findings are consistent with a study from Turkey and Nigeria, which reported low acceptance rates for gamete donation to infertile women when necessary for treatment, and contrast with an Iranian study, where a significant number of people accepted surrogacy [36–38]. In Lebanon, the presence of various religious communities has contributed to a significant legislative gap in laws and regulations related to acceptance or refusal of gamete donation and gestational surrogacy [39,40].

Analysis of the associated factors revealed that women who received information about ART from a doctor had more positive attitudes. This is evident in other studies, where higher levels of healthcare support and psychosocial care are shown to reduce stress, improve lifestyle outcomes, fertility-related knowledge, patient well-being, and treatment compliance [41]. Additionally, participants in rural areas were found to have higher ART attitude scores compared to those in central areas, which contrasts with findings in Iran [28]. This difference may be attributed to the perception of infertility as a more pressing issue in rural areas, leading to greater acceptance of ART as a viable solution. Moreover, older women demonstrated more positive attitudes toward ART, likely because they may turn to ART as a source of hope for having a child. These results contrast with other findings suggesting that generational differences or societal attitudes toward ART may vary across different populations [28].

Regarding expectations of ART, most participants had high hopes, likely due to the strong desire for motherhood. Notably, no existing studies specifically evaluate expectations related to ART. While the majority did not expect ART procedures to be easy or always successful, the majority believed that these procedures would not be accessible or affordable in our country. Such perception aligns with findings from studies conducted in Nigeria, Iran, and Ghana [27,28,42]. Besides, more than half of the participants believed that pregnancy through ART would be the same as natural conception and that ART procedures would be safe for both women and fetuses. These findings align with a Hungarian study, where most participants were unaware of ART’s potential side effects on both the mother and fetus [26]. The search for the associated factors revealed that patient expectations differ across various fertility clinics, underscoring the variability in perspectives, treatment approaches, and communication strategies. These factors can significantly influence how patients perceive and anticipate their experiences with ART. This aligns with findings from a meta-analysis, which emphasizes that a lack of compassion in the healthcare environment, coupled with poor communication, negatively impacts women’s experiences with infertility and treatment [43].

This study aimed to assess the knowledge, attitudes, and expectations towards ART among Lebanese women experiencing difficulty conceiving. Yet, several limitations should be considered. The study’s cross-sectional design cannot establish causality, but only generate hypotheses. Although women were selected from two Beirut clinics, they were from various Lebanese regions, with an over-representation from the Mount Lebanon area. This may introduce selection bias and limit the generalizability of the findings. Information bias is another concern, as the sensitive nature of some questions could lead to underreporting. Over-reporting might also occur, as women who are already willing to undergo ART may have more knowledge, optimism, and higher expectations. Social desirability bias is particularly relevant given the sensitive ART topics and the face-to-face interview format. Finally, the absence of test–retest reliability assessment may limit the reproducibility of the questionnaire.

## Conclusion

This study highlights important sociodemographic, clinical, and behavioral factors associated with knowledge, attitudes, and expectations toward ART among women facing infertility in Lebanon. While many women demonstrate good understanding, positive attitudes, and expectations toward ART, these are shaped by several factors such as age, education, medical history, lifestyle, and socioeconomic conditions. Recognizing these influences can help clinicians and policymakers address gaps in reproductive health services through targeted interventions, including integrating ART education into primary care, overcoming financial and accessibility barriers, providing psychological support, and tailoring a personalized, holistic approach. Due to the study design and the sensitive nature of the topic, the findings cannot be generalized to the broader population. Therefore, future research should further explore how socioeconomic, geographic, and psychosocial factors affect ART outcomes and assess the knowledge in the general population to facilitate early support. This also underscores the need for collaborative legal frameworks to address ethical and social challenges related to ART.

## Submission declaration and verification

This manuscript is not under consideration for publication elsewhere.

All authors have approved the manuscript for submission, and the research has received approval from the institutional review board of the School of Pharmacy at the Lebanese University and the Clinical Research Unit at Al Zahraa Hospital University Medical Center. If accepted, this article will not be published elsewhere in the same form, in English or any other language, without written consent from the copyright-holder.

## Supporting information

S1 Data Dataset. (XLSX)
